# Are negative symptoms evaluated with a self-assessment tool or a semi-structured interview more strongly correlated to mental health-related quality of life?

**DOI:** 10.1192/j.eurpsy.2025.2240

**Published:** 2025-08-26

**Authors:** J. Montvidas, E. Zauka, Ž. Žumbakys, V. Adomaitienė

**Affiliations:** 1 Psychiatry, Lithuanian University of Health Sciences, Kaunas, Lithuania

## Abstract

**Introduction:**

Negative symptoms are one of the core symptom groups of schizophrenia. These symptoms are highly prevalent and are proven to have a strong correlation with mental health-related quality of life. Evaluating negative symptoms using the Brief Negative Symptoms Score (BNSS), a semi-structured interview, is recommended. BNSS can be supplemented with the Self-assessment of Negative Symptoms (SNS), a self-assessment scale. It is unclear whether a semi-structured interview or a self-assessment scale is more related to the mental health-related quality of life.

**Objectives:**

To evaluate whether scores BNSS or SNS are more strongly correlated with the mental health-related quality of life.

**Methods:**

We performed a cross-sectional study in an inpatient clinic of a university hospital in Lithuania. Inclusion criteria were a diagnosis of schizophrenia spectrum disorder according to ICD-10, age between 18 and 65. Exclusion criteria were acute and/or severe comorbid psychiatric or somatic disorders. BNSS and SNS were used to evaluate negative symptoms. The 36-item Short Form survey (SF-36) was used to evaluate quality of life. Three independent psychiatrists evaluated the participants of the study. The first psychiatrist evaluated the negative symptoms with BNSS. The second psychiatrist handed out, collected, and scored SNS. The third psychiatrist handed out, collected, and scored SF-36. Afterward, the statistical correlation analysis was performed. Only the energy/fatigue and mental health subscores of SF-36 were included in the study to limit the correlation analysis to only the mental health-related quality of life.

**Results:**

The study included 93 participants. We found that SNS scores significantly correlated with mental health-related quality of life compared. SNS had higher correlation indexes with the energy/fatigue subscore than the mental health subscore of SF-36. The strongest correlation was seen between the total score of SNS and the energy/fatigue subscore of SF-36 (r=-0,508, p<0,001). BNSS had no statistically significant correlations with either the energy/fatigue or the mental health subscore of SF-36. All of the correlation coefficients can be seen in Table 1.

**Table:** 

Variable	Correlation coefficient MH	p-value	Correlation coefficient EF	p-value
SNS SI	-0,294	0,04	-0,358	<0,001
SNS A	-0,258	0,13	-0,251	0,015
SNS AV	-0,26	0,012	-0,398	<0.001
SNS AN	-0,354	<0.001	-0,434	<0.001
SNS TS	-0,348	<0,001	-0.508	<0,001

Table 1. Correlation coefficients of SNS and SF-36 scores. MH – SF-36 mental health subscore; EF- SF-36 energy/fatigue subscore; SNS SI – SNS social isolation subscore, SNS A- SNS alogia subscore; SNS AV – SNS avolition subscore; SNS AN – SNS anhedonia subscore; SNS total score.

**Conclusions:**

SNS, a self-evaluation scale, was more strongly correlated to mental health-related quality of life than scores of BNSS.

**Disclosure of Interest:**

None Declared

